# Epidemiology and Survival Outcomes for Eyelid Primary Malignant Melanoma: An Analysis of 1397 Cases in the SEER Database

**DOI:** 10.1155/2020/4858636

**Published:** 2020-12-08

**Authors:** Yi Shan, Yufeng Xu, Yuexin Lu, Menglu Chen, Jing Cao, Yijie Wang, Xiling Lin, Juan Ye

**Affiliations:** ^1^Department of Ophthalmology, The Second Affiliated Hospital of Zhejiang University, College of Medicine, 88 Jiefang Road, Hangzhou, Zhejiang 310009, China; ^2^Department of Surgical Oncology, The Second Affiliated Hospital of Zhejiang University, College of Medicine, 88 Jiefang Road, Hangzhou, Zhejiang 310009, China; ^3^Department of Endocrinology, The Second Affiliated Hospital of Zhejiang University, School of Medicine, 88 Jiefang Road, Hangzhou, Zhejiang 310009, China

## Abstract

**Purpose:**

There has not been a recent population-based study regarding the epidemiological trend and survival of eyelid primary malignant melanoma (PMM). Our study aims to evaluate the updated incidence trends and discuss the factors affecting the survival outcomes of eyelid PMM.

**Methods:**

A total of 1397 eyelid PMM cases diagnosed between 1975 and 2016 were retrospectively identified from the Surveillance, Epidemiology, and End Results (SEER) database. Age-adjusted incidence rates and annual percent changes (APC) were calculated. Kaplan-Meier and Cox proportional hazards regression models were used to calculate survival outcomes and identify potential prognostic factors.

**Results:**

The overall age-adjusted incidence of eyelid PMM rose from 0.039 (95% confidence interval [CI], 0.012–0.088) in 1975 to 0.103 (95% CI, 0.070–0.143) per 100 000 population in 2016, with significant APC of 1.313% (*p* < 0.001). Male subjects showed a higher average age-adjusted incidence rate than female subjects (*p* < 0.001). Survival analyses showed that 5-year accumulative overall survival (OS) and disease-specific survival (DSS) for patients with eyelid PMM were 70.5% and 90.6%. Additionally, 10-year OS and DSS were 51.8% and 86.1%, respectively. Analyses of Kaplan-Meier survival curves with the log-rank test revealed that older age, White race, nodular melanoma, higher American Joint Committee on *Cancer* (AJCC) stage (II to IV), advanced stage, distant metastasis, and no-surgery treatment were associated with lower OS and DSS rates. Age, histology, AJCC stage, and stage at diagnosis were found to be independent predictors of OS and DSS in multivariate models.

**Conclusion:**

The incidence of eyelid PMM increased with significant APC and male predominance. Age, histology, AJCC stage, and stage at diagnosis might be independent predictors of prognosis, emphasizing the importance of improved diagnosis of eyelid PMM.

## 1. Introduction

Eyelid primary malignant melanoma (PMM) is a rare and aggressive cancer of melanocytes, producing the pigment melanin [1,2]. Eyelid PMM accounts for approximately 1% of all cutaneous melanomas, representing less than 1% of all eyelid malignancies [3,4]. Despite low proportion, melanoma of eyelid skin is associated with two-thirds of tumor-related deaths from eyelid malignancies [5]. Because of the rarity of eyelid PMM, previous publications about cutaneous eyelid melanomas were a few retrospective studies and case reports with a small sample size [2,6–9].

These limited studies showed the risk factors of eyelid PMM, including changing abnormal skin nevi, excessive exposure to sunlight, family history of melanoma, and light-skinned population [1,3,4,9–12]. Eyelid PMM was typically diagnosed in the elderly, rarely seen among the young population [3]. Lentigo malignant melanoma (LMM) and superficial spreading melanoma (SSM) represented the two largest histologic subgroups [6,13]. Wide surgical excision has been recommended as the first-line therapy for cutaneous eyelid melanomas. Patients with eyelid PMM at diagnosis mostly met the American Joint Committee on Cancer (AJCC) stage I criteria [6]. Vaziri et al. [9] reported that eyelid PMM had a relatively good clinical prognosis. Although melanoma of eyelid skin is almost initially diagnosed by ophthalmologists, unfamiliarity with the clinical characteristics increases the risk of misdiagnosis and underdiagnosis.

There is not yet a comprehensive population-based study evaluating the epidemiological trends, clinicopathological features, and survival outcomes of eyelid PMM. The Surveillance, Epidemiology, and End Results (SEER) Program of the National Cancer Institute (NCI) provides authoritative and extensive data on incidence and survival outcomes of rare malignancies in the United States. In this study, we aimed to provide analyses of eyelid PMM in terms of updated epidemiological trends and prognostic clinicopathological factors in the United States, using the SEER resource.

## 2. Methods

### 2.1. Study Design

A population-based retrospective analysis for patients with eyelid PMM was conducted using the SEER 18 Registries Database (http://www.seer.cancer.gov). The SEER Program is one of the largest and most authoritative sources of the cancer-related dataset in the United States, which is sponsored by the US NCI. The SEER database collects cancer incidence, patients' clinicopathological features, and survival data from 18 population-based cancer registries, covering 28% of the US population, including 23% of African Americans and 40% of Hispanics.

### 2.2. Data Collection

Cases with eyelid PMM from 1975 to 2016 were selected according to the *International Classification of Diseases for Oncology, Third Edition (ICD-O-3)*, morphology codes (8720/3–8790/3), and site-specific code *C44.1* [14–16]. The inclusion criteria of survival analysis were positive histology, complete survival month, and active follow-up records. We excluded patients only confirmed by autopsy or death certificate.

Variables such as age at diagnosis, gender, race (White, Black, American Indian/AK Native (AIAN), and Asian/Pacific Islander (API)), origin (Non-Hispanic and Hispanic), year of diagnosis, primary laterality (left, right, bilateral, and side unspecified), histological type, AJCC stage, stage at diagnosis (localized, regional, and distant), metastasis, surgery, radiation, and chemotherapy were extracted [17]. Histologic characteristics were categorized as melanoma not otherwise specified (NOS), Lentigo malignant melanoma (LMM), superficial spreading melanoma (SSM), nodular melanoma (NM), and others. “Malignant melanoma, NOS” indicates no tumor subtype in patient records. There was an increasing incidence trend of head and neck melanoma in the younger population (aged 0–39 years) in America [14]. Based on these findings, we divided the age into four age groups (0–39, 40–59, 60–79, and 80+ years).

### 2.3. Statistical Analysis

Age-adjusted incidence rates (AAIRs) were presented as cases per 100 000 persons using 2000 US Standard Population as a reference population [18].

AAIRs and annual percent change (APC) were calculated via SEER ∗ Stat software version 8.3.6 (National Cancer Institute, Rockville, Maryland). The overall survival (OS) and disease-specific survival (DSS) were calculated using the Kaplan-Meier method. The log-rank test was applied to test the OS and DSS differences between different subgroups. Multivariate Cox analysis was conducted utilizing Cox proportional hazards regression to identify the prognostic predictors of OS and DSS. Statistical analyses and graphics were conducted using IBM SPSS 25.0 Statistical Software (SPSS, Inc, Chicago, IL) and Prism Software (version 8; GraphPad). A *p* value of *<* 0.05 was considered to be statistically significant.

## 3. Results

### 3.1. Patient Characteristics

A total of 1397 patients with melanoma of eyelid skin were finally identified. The general demographic and clinicopathological characteristics of this cohort are summarized in Table 1. The median age at diagnosis of patients was 71.0 ± 16.8 years (range, 4–103 years). Melanoma of eyelid appeared to remain more common in males (740 subjects, 53.0%) than females (657 subjects, 47.0%) (*p*=0.028). White patients (95.6%) made up the majority, followed by Asian or Pacific Islander (1.0%), Blacks (0.3%), and American Indian/Alaska Native (0.3%). The entire cohort was composed of 94.1% Non-Hispanics and 5.9% Hispanics. Malignant melanoma occurred by 52.1% on the left eyelid and 46.2% on the right eyelid. Malignant melanoma, NOS represented 47.8% of all melanomas, followed by LMM (20.5%), SSM (18.6%), and NM (7.3%). The majority of these cases were diagnosed at early AJCC stage I (34.5%) and localized stage (77.7%). The AJCC TNM staging data was listed in Table S1 in the Supplementary Materials. Surgery was performed in 93.1% of patients. Radiation therapy and chemotherapy were, respectively, performed in 3.1% and 1.0% patients.

### 3.2. Incidence Analysis

**Figure 1:**
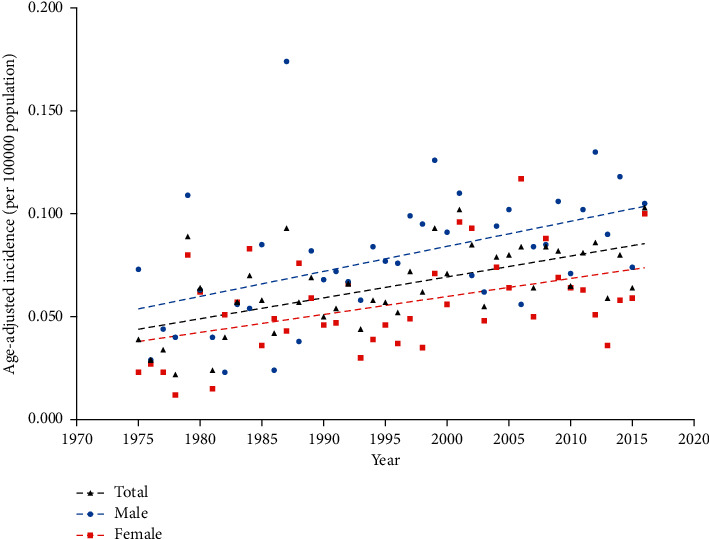
Age-adjusted incidence of eyelid PMM by sex from 1975 to 2016. Linear Regression for trends in age-adjusted incidence (per 100 000 population) of eyelid PMM among males and females. PMM, primary malignant melanoma.

Total and gender-specific incidence rates increased steadily over time (Figure 1). The overall age-adjusted incidence of malignant melanoma of the eyelid rose from 0.039 per 100 000 population in 1975 to 0.103 per 100 000 population in 2016, with an APC of 1.313% (95% confidence interval [CI], 0.635–1.995%; *p* < 0.001). Similar rising temporal patterns were observed in both males (APC, 1.205%, *p*=0.007) and females (APC, 1.316%, *p*=0.012) from 1975 to 2016. Male subjects showed a higher average age-adjusted incidence rate than female subjects (*p* < 0.001).

### 3.3. Survival and Univariate Analysis

**Figure 2:**
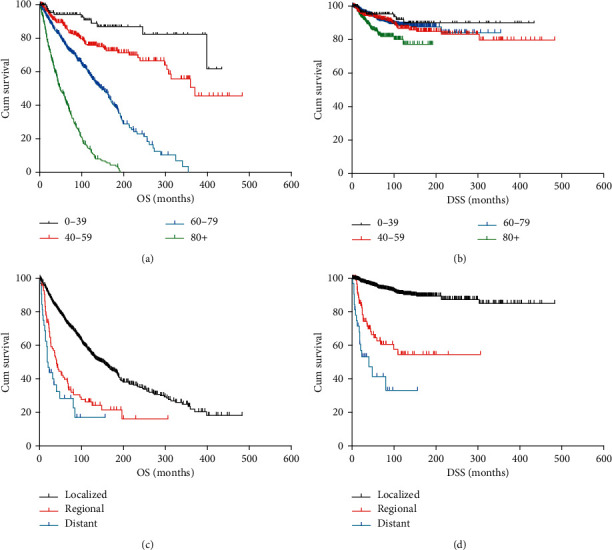
Kaplan-Meier survival analysis for each group: (a) OS for patients with eyelid PMM by age range; (b) DSS for patients with eyelid PMM by age range; (c) OS for patients with eyelid PMM by stage at diagnosis; (d) DSS for patients with eyelid PMM by stage at diagnosis. PMM, primary malignant melanoma; OS, overall survival; DSS, disease-specific survival.

The median follow-up of the study was 127 months (range, 0 to 483 months). 137 patients (9.8%) died from melanoma of the skin during the period. 5-year accumulative OS and DSS for eyelid malignant melanoma were 70.5% and 90.6%, respectively. Additionally, 10-year OS and DSS were 51.8% and 86.1%, respectively. The OS and DSS analyses according to the demographic and clinicopathological characteristics are presented in Table 2. Both OS and DSS analyses of Kaplan-Meier survival curves with the log-rank test revealed significant poorer survival rates in old patients (OS: 40–59, *p*=0.005, 60–79, *p* < 0.001, 80+, *p* < 0.001; DSS: 80+, *p*=0.007) (Figures 2(a) and 2(b)). Besides, a higher stage of tumor was linked with lower survival rates (OS: regional, *p* < 0.001, distant, *p* < 0.001; DSS: regional, *p* < 0.001, distant, *p* < 0.001) (Figures 2(c) and 2(d)). Analyses revealed that SSM (OS: *p*=0.003; DSS: *p*=0.006) was in connection with higher OS and DSS rates. NM (OS: *p*=0.001; DSS: *p*=0.004), advanced AJCC stage (OS : II, *p* < 0.001, III, *p* < 0.001, IV*p* < 0.001; DSS : II, *p* < 0.001, III, *p* < 0.001, IV, *p* < 0.001), distant metastasis (OS: *p* < 0.001; DSS: *p* < 0.001), and no-surgery treatment (OS: *p* < 0.001; DSS: *p*=0.009) showed lower OS and DSS rates. Moreover, OS of White patients was significantly lower than that of other races (*p*=0.009). LMM (*p* < 0.001), SSM (*p*=0.006), no-radiation (*p* < 0.001), and no-chemotherapy group (*p* < 0.001) indicated higher DSS rates.

### 3.4. Multivariate Analysis

We analyzed the independent effects of prognostic factors using the multivariate Cox regression analysis model (Table 3). In the OS analysis, older age (40–59, HR 2.623, 95% CI 1.387–4.960, *p* = 0.003; 60–79, HR 7.530, 95% CI 4.072–13.926, *p* < 0.001; 80+, HR 24.216, 95% CI 13.038–44.976, *p* < 0.001), higher AJCC stage (II, HR 1.602, 95% CI 1.142–2.246, *p*=0.006; III, HR 1.885, 95% CI 1.277–4.051, *p* < 0.001; IV, HR 6.183, 95% CI 3.158–12.104, *p* < 0.001), and advanced stage at diagnosis (regional, HR 1.983, 95% CI 1.497–2.628, *p* < 0.001; distant, HR 3.906, 95% CI 2.277–6.701, *p* < 0.001) were independent predictors of poorer prognosis. In the DSS analysis, age over 80 (HR 43.707, 95% CI 1.640–8.381, *p*=0.002), advanced stage (regional, HR 4.540, 95% CI 2.781–7.411, *p* < 0.001; distant, HR 13.520, 95% CI 6.870–26.604, *p* < 0.001), and higher AJCC stage (II, HR 3.738, 95% CI 1.869–7.476, *p* < 0.001; III, HR 5.397, 95% CI 1.754–16.613, *p*=0.003; IV, HR 22.570, 95% CI 8.589–59.312, *p* < 0.001) were found to be independent negative predictors of DSS. Additionally, LMM (HR 0.243, 95% CI 0.117–0.506, *p* < 0.001) and SSM (HR 0.514, 95% CI 0.299–0.884, *p*=0.016) were prognostic indicators of increased DSS rate.

## 4. Discussion

This current study reported a large cohort of eyelid PMM. A total of 1397 patients in the SEER database diagnosed between 1975 and 2016 were finally identified, so our results are probably more reliable. The incidence of eyelid PMM increased with significant APC and male predominance. Age, histology, AJCC stage, and stage at diagnosis might be independent predictors of prognosis.

According to our study, the overall age-adjusted incidence increased over the past 4 decades with a significantly higher incidence in males. Cutaneous eyelid melanoma accounts for a small proportion of the estimated 96,480 new cases of cutaneous melanoma in the United States in 2019 [19,20]. The rising incidence of cutaneous melanoma was reported by Paulson et al. [21] and Yang et al. [22]. Previous studies reported the age-adjusted incidence of eyelid melanoma: 0.08 cases per 100 000 individuals per year from 1976 through 1990 by Cook et al. 0.6 per million Whites older than 20 years in the 1990s by Margo et al. 0.1 per 100 000 overall in Ireland from 2005 to 2015 by Quigley et al. [8,23,24]. The rising incidence rate in America may be due to inadequate efforts to take measures for sun protection (e.g., sunglasses) and population growth.

Sex-related incidence patterns by year were reported in this study. The male predominance of the average incidence rate in our study was in agreement with previous research on head and neck melanoma in the US and Canada [14]. Also, Oliver et al. [7] found that the male: female ratio in eyelid melanoma was 1.08. The reasons for gender differences might be attributed to the excessive ultraviolet (UV) exposure of men and careful daily skin check of women. Patel et al. [25] revealed that men tend to have more opportunities to do the outdoor jobs and activities (e.g., the construction industry, sports, and farming). However, gender was not a significant prognostic indicator of survival in our study. This is different from a previous study, which found that men with cutaneous melanomas had a significant survival disadvantage (*p*=0.02) [4].

In this cohort, the 5-year OS rate and DSS rate of eyelid PMM were 70.5% and 90.6%, respectively. Furthermore, 10-year OS and DSS were 51.8% and 86.1%, respectively. This clinical outcome was similar to that of another report [9]. A study by Xu Y et al. [26] revealed that 5-year OS and DSS for uveal melanoma were 61.8% and 66.5%, respectively. In univariate analysis, the White race, older age, NM, higher AJCC stage (II to IV), advanced stage at diagnosis, distant metastasis, and no-surgery treatment were associated with significantly lower OS and DSS rates. However, only factors of age, histology, AJCC stage, and stage at diagnosis were found to be independent predictors in multivariate analyses.

We identified that eyelid PMM had some similar clinicopathological features, compared with previous studies [3,9,10]. Eyelid PMM affected all ages (range, 4–103 years) but is most common in elderly adults. The median age at diagnosis of our study population was 71.0 years, which is higher than the mean age of 64–68 years reported in previous studies [3,9,24,27]. Older patients (80+) had a significantly worse OS and DSS rate than the younger patients. We hypothesize that the eyelid skin is chronically stimulated by physical and chemical factors such as UV radiation [28], which is directly exposed to the external environment. Furthermore, the patients usually presented with a painless and pigmented eyelid skin mass that might be ignored as a pigmented nevus or a birthmark [29]. Therefore, the diagnosis and treatment may be delayed until the older age. Eyelid PMM mainly affects White patients (95.6%). Epidemiological data revealed that Black individuals had a lower incidence of skin cancer compared to White individuals [30]. It has been proved that melanin has important roles in photoprotection [31]. LMM was the most common histological type of eyelid PMM in previous studies [3,9,32]. Among 1397 patients in this cohort, LMM was found to be the most common subtype excluding malignant melanoma, NOS. Garner et al. [13] revealed that cutaneous superficial subtypes resulted in the relatively better prognosis of eyelid melanoma [13]. The worst survival outcomes of NM can be partly associated with a vertical growth trend and quick metastatic progression [33].

While the SEER database was lacking further detailed AJCC staging, we identified the AJCC stage as a significant prognostic factor for patients with eyelid PMM. The multivariate analysis proved that AJCC II to IV stage was an independent prognostic factor for worse OS and DSS. Isaksson et al. [34] reported that 5-year and 10-year melanoma-specific survivals for stage III cutaneous malignant melanoma were 59% and 51%, respectively. We also observed that distant metastasis is associated with poor prognosis compared with the localized stage. Due to the limitation of a small sample size of previous studies, subsequent studies are needed to confirm the conclusion from multiple data sources.

As is well known, the main treatment of PMM was complete surgical excision [9,12]. In our study, patients who underwent surgery had improved survival period than the no-surgery group in univariate analyses. A previous study found that a minimum surgical excision margin for eyelid melanoma of ≤1 mm in Breslow thickness was 3 mm [4]. The no-radiation and no-chemotherapy had a better DSS rate in univariate analyses. Patients with lower AJCC stage were likely to conduct surgery instead of radiation or chemotherapy.

This study is a comprehensive population-based analysis of eyelid PMM in a large cohort using the latest updated SEER database. The SEER database provides an incomparable source when investigating rare cancers. We have easy access to large-scale data from multiple centers of SEER registries.

The present study had several limitations. First, the outcome of some statistical tests needs to be confirmed by further studies because of the small sample size compared to other tumor-related researches. Previous research found histological subtype was an independent prognostic factor for melanoma [35]. However, the high proportion of malignant melanoma NOS in our study indicates that the SEER database does not contain sufficient data on a specific histological diagnosis. This reduces the veracity to clarify histological diagnosis related to survival. Second, over 70% of the patients were with unknown status of metastasis at diagnosis. The possible reason might be lack of data records of earlier cases in the SEER database. Furthermore, more sun exposure of the lower eyelid and sun protection on the brows above the upper eyelid caused the lower eyelid to be more affected [3]. Nevertheless, the SEER database does not include some information about the location on upper or lower lids, detailed surgical depiction, comorbidities, family history, and tumor recurrence. Additionally, the study is carried out retrospectively. The selective bias related to collected data is inevitable. There are many censored data, which can be supplemented in future research. Thus, long-term studies are needed to verify our conclusion.

## 5. Conclusion

In conclusion, we showed that the overall incidence rate of eyelid PMM had risen from 1975 to 2016 with an APC of 1.313%. The gender tendency has existed since 1990. Our study reported that age, histology, AJCC stage, and stage at diagnosis were significantly associated with a worse prognosis. These findings might help ophthalmologists guide clinical decision making in early-stage diagnosis and management of eyelid PMM.

## Figures and Tables

**Table 1:** Demographic and clinicopathological characteristics of patients with eyelid PMM, 1975–2016.

Characteristic	*N*	%
Total	1397	100

*Age at diagnosis, y*		
0–39	94	6.7
40–59	309	22.1
60–79	596	42.7
80+	398	28.5

*Gender*		
Male	740	53.0
Female	657	47.0

*Race*		
White	1335	95.6
Black	4	0.3
Asian or pacific islander	14	1.0
American Indian/Alaska native	5	0.3
Unknown	39	2.8

*Ethnicity*		
Hispanic	83	5.9
Non-Hispanic	1314	94.1

*Laterality*		
Left	727	52.1
Right	646	46.2
Bilateral	9	0.6
Side unspecified	15	1.1

*Histologic subtype*		
8720/3: Malignant melanoma, NOS	668	47.8
8742/3: LMM	287	20.5
8743/3: SSM	260	18.6
8721/3: NM	102	7.3
8745/3: Desmoplastic melanoma	28	2.0
8772/3: Spindle cell melanoma, NOS	22	1.6
8730/3: Amelanotic melanoma	12	0.9
8771/3: Epithelioid cell melanoma	7	0.5
8740/3: Malignant melanoma in junctional nevus	2	0.1
8744/3: Acral lentiginous melanoma	2	0.1
8770/3: Mixed epithelioid and spindle cell melanoma	2	0.1
8722/3: Balloon cell melanoma	1	0.1
8723/3: Malignant melanoma, regressing	1	0.1
8741/3: Malignant melanoma in precancerous melanosis	1	0.1
8761/3: Malignant melanoma in giant pigmented nevus	1	0.1
8773/3: Spindle cell melanoma, type A	1	0.1

*AJCC stage*		
I	482	34.5
II	112	8.0
III	19	1.4
IV	15	1.1
Unknown	769	55.0

*Stage at diagnosis*		
Localized	1085	77.7
Regional	98	7.0
Distant	32	2.3
Unknown	182	13.0

*Metastasis at diagnosis*		
None	398	28.5
Distant	10	0.7
Unknown	989	70.8

*Surgery*		
Yes	1301	93.1
No	96	6.9

*Radiation Chemotherapy*		
Yes	43	3.1
No	1354	96.9

*Chemotherapy*		
Yes	14	1.0
No	1383	99.0

PMM, primary malignant melanoma; NOS, not otherwise specified; LMM, lentigo malignant melanoma; SSM, superficial spreading melanoma; NM, nodular melanoma; AJCC, American Joint Committee on Cancer.

**Table 2:** Univariate analysis of factors affecting 5-year and 10-year OS and DSS of eyelid PMM.

Characteristics	OS		Log rank	DSS		Log rank
	5-year	10-year	*p* value	5-year	10-year	*p* value
Overall	70.5	51.8		90.6	86.1	

*Sex*						
Male	67.3	49.4	Reference	89.5	86.2	Reference
Female	74.1	54.4	0.068	99.2	97.6	0.317

*Race*						
White	69.8	50.8	Reference	90.2	85.7	Reference
Others	90.1	81.1	**0.009** ^*∗∗*^	100	96.0	0.052

*Ethnicity*						
Hispanic	72.7	63.7	Reference	91.9	89.3	Reference
Non-Hispanic	70.4	51.1	0.542	90.5	85.9	0.714

*Age, y*						
<40	94.2	90.9	Reference	95.4	92.0	Reference
40–59	86.8	75.7	**0.005** ^*∗∗*^	93.0	86.6	0.186
60–79	76.0	57.6	**<0.001** ^*∗∗∗*^	91.8	88.5	0.342
80+	43.9	13.7	**<0.001** ^*∗∗∗*^	84.3	79.9	**0.007** ^*∗∗*^

*Laterality*						
Left	72.2	53.4	Reference	91.6	88.0	Reference
Right	69.5	50.8	0.501	89.2	83.9	0.190

*Histology*						
Malignant melanoma, NOS	69.3	51.8	Reference	88.8	84.8	Reference
LMM	73.5	54.2	0.692	99.0	94.8	**<0.001** ^*∗∗∗*^
SSM	82.1	58.8	**0.003** ^*∗∗*^	95.4	89.2	**0.006** ^*∗∗*^
NM	56.5	36.7	**0.001** ^*∗∗*^	76.4	71.5	**0.004** ^*∗∗*^

*AJCC stage*						
I	79.3	61.1	Reference	97.3	93.9	Reference
II	64.4	53.7	**<0.001** ^*∗∗∗*^	80.8	76.6	**<0.001** ^*∗∗∗*^
III	57.0	46.0	**<0.001** ^*∗∗∗*^	75.9	N/A	**<0.001** ^*∗∗∗*^
IV	40.0	20.0	**<0.001** ^*∗∗∗*^	50.3	N/A	**<0.001** ^*∗∗∗*^

*Stage at diagnosis*						
Localized	75.9	56.1	Reference	94.7	90.8	Reference
Regional	39.8	26.2	**<0.001** ^*∗∗∗*^	62.7	54.5	**<0.001** ^*∗∗∗*^
Distant	28.4	17.0	**<0.001** ^*∗∗∗*^	41.4	33.1	**<0.001** ^*∗∗∗*^

*Metastasis at diagnosis*						
None	72.1	N/A	Reference	92.9	N/A	Reference
Distant	40.0	N/A	**<0.001** ^*∗∗∗*^	50.0	N/A	**<0.001** ^*∗∗∗*^

*Surgery*						
Yes	71.9	53.1	Reference	91.1	86.6	Reference
No	60.7	28.6	**<0.001** ^*∗∗∗*^	83.6	77.2	**0.009** ^*∗∗*^

*Radiation chemotherapy*						
Yes	59.9	37.9	Reference	74.2	49.5	Reference
No	71.5	52.2	**<0.001** ^*∗∗∗*^	91.1	87.3	**<0.001** ^*∗∗∗*^

*Chemotherapy*						
Yes	69.6	46.4	Reference	77.4	46.4	Reference
No	70.6	51.8	0.425	90.8	86.6	**<0.001** ^*∗∗∗*^

OS, overall survival; DSS, disease-specific survival; PMM, primary malignant melanoma; NOS, not otherwise specified; LMM, lentigo malignant melanoma; SSM, superficial spreading melanoma; NM, nodular melanoma; AJCC, American Joint Committee on Cancer. Bold letters indicate statistical significance compared with references (*p* < 0.05). ^*∗*^*p* < 0.05; ^*∗∗*^*p* < 0.01; ^*∗∗∗*^*p* < 0.001.

**Table 3:** Multivariate cox proportional hazard regression analysis of prognostic factors for OS and DSS of patients with eyelid PMM.

Characteristics	OS		DSS	
	HR (95% CI)	*p* value	HR (95% CI)	*p* value
*Race*		0.232		0.196
Others	Reference		Reference	
White	0.825 (0.601–1.131)	0.232	0.515 (0.188–1.409)	0.196

*Age, y*		**<0.001** ^*∗∗∗*^		**<0.001** ^*∗∗∗*^
0–39	Reference		Reference	
40–59	2.623 (1.387–4.960)	**0.003** ^*∗∗*^	2.022 (0.887–4.608)	0.094
60–79	7.530 (4.072–13.926)	**<0.001** ^*∗∗∗*^	1.823 (0.818–4.062)	0.142
80+	24.216 (13.038–44.976)	**<0.001** ^*∗∗∗*^	3.707 (1.640–8.381)	**0.002** ^*∗∗*^

*Histology*		0.054		**<0.001** ^*∗∗∗*^
Malignant melanoma, NOS	Reference		Reference	
LMM	0.809 (0.652–1.004)	0.055	0.243 (0.117–0.506)	**<0.001** ^*∗∗∗*^
SSM	0.828 (0.656–1.046)	0.113	0.514 (0.299–0.884)	**0.016** ^*∗*^
NM	1.192 (0.893–1.582)	0.234	1.531 (0.921–2.545)	0.100

*AJCC stage*		**<0.001** ^**∗∗∗**^		**<0.001** ^*∗∗∗*^
I	Reference		Reference	
II	1.602 (1.142–2.246)	**0.006** ^*∗∗*^	3.738 (1.869–7.476)	**<0.001** ^*∗∗∗*^
III	1.885 (1.277–4.051)	**<0.001** ^*∗∗∗*^	5.397 (1.754–16.613)	**0.003** ^*∗∗*^
IV	6.183 (3.158–12.104)	**<0.001** ^*∗∗∗*^	22.570 (8.589–59.312)	**<0.001** ^*∗∗∗*^

*Stage at diagnosis*		**<0.001** ^*∗∗∗*^		**<0.001** ^*∗∗∗*^
Localized	Reference		Reference	
Regional	1.983 (1.497–2.628)	**<0.001** ^*∗∗∗*^	4.540 (2.781–7.411)	**<0.001** ^*∗∗∗*^
Distant	3.906 (2.277–6.701)	**<0.001** ^*∗∗∗*^	13.520 (6.870–26.604)	**<0.001** ^*∗∗∗*^

*Metastasis at diagnosis*		0.869		0.963
None	Reference		Reference	
Distant	1.440 (0.370–5.614)	0.599	0.836 (0.130–5.381)	0.851

*Surgery*		0.361		0.520
No	Reference		Reference	
Yes	0.866 (0.637–1.179)	0.361	0.821 (0.451–1.496)	0.520

HR, hazard ratio; CI, confidence interval; PMM, primary malignant melanoma; NOS, not otherwise specified; LMM, lentigo malignant melanoma; SSM, superficial spreading melanoma; NM, nodular melanoma; OS, overall survival; DSS, disease-specific survival; AJCC, American Joint Committee on Cancer. Bold letter indicates statistical significance compared with references (*p* < 0.05). ^*∗*^*p* < 0.05; ^*∗∗*^*p* < 0.01; ^*∗∗∗*^*p* < 0.001.

## Data Availability

The raw data about eyelid PMM cases supporting this research are from http://www.seer.cancer.gov. The data used to support the findings of this study are available from the corresponding author upon request (yejuan@zju.edu.cn).

## Supplementary Materials

Supplementary Materials
Table S1: TNM characteristics of patients with eyelid PMM, 1975–2016.
