# Supplementary material for: Epidemiology and Survival Outcomes for Eyelid Primary Malignant Melanoma: An Analysis of 1397 Cases in the SEER Database
Source: J Ophthalmol. 2020 Dec 8;2020:4858636. doi: 10.1155/2020/4858636 (PMC7803156; doi:10.1155/2020/4858636)
Supplement: Supplementary Materials — Table S1: TNM characteristics of patients with eyelid PMM, 1975–2016. [file 4858636.f1.docx]

**Supplemental files**

Table S1: TNM characteristics of patients with eyelid PMM, 1975-2016.

| **Characteristic** | N | % |
| --- | --- | --- |
| **Tumor depth/Breslow’s thickness, mm** |  |  |
| T0 | 1 | 0.1 |
| T1 | 453 | 32.4 |
| T2 | 100 | 7.2 |
| T3 | 68 | 4.9 |
| T4 | 45 | 3.2 |
| Tx | 135 | 9.6 |
| Blank | 595 | 42.6 |
| **N stage** |  |  |
| N0 | 704 | 50.5 |
| N1 | 16 | 1.1 |
| N2 | 6 | 0.4 |
| N3 | 2 | 0.1 |
| Nx | 74 | 5.3 |
| Blank | 595 | 42.6 |
| **M stage** |  |  |
| M0 | 727 | 52.0 |
| M1 | 15 | 1.1 |
| MX | 60 | 4.3 |
| Blank | 595 | 42.6 |

Breslow’s thickness was categorized according to AJCC TNM staging as 0.01-1.00 mm, 1.01-2.00 mm, 2.01-4.00 mm, and >4.00mm. TNM, Tumor Node Metastasis.
